# Research on Factors Affecting the Entrepreneurial Learning From Failure: An Interpretive Structure Model

**DOI:** 10.3389/fpsyg.2019.01304

**Published:** 2019-06-04

**Authors:** Jiangru Wei, Yuting Chen, Jing Zhang, Yonghua Gong

**Affiliations:** School of Management, Nanjing University of Posts and Telecommunications, Nanjing, China

**Keywords:** entrepreneurial learning from failure, ISM, entrepreneurial education, self-efficacy, emotion regulation

## Abstract

Based on the interpretive structure model of system dynamics, this paper constructs a hierarchical structure model of factors affecting the entrepreneurial learning from failure, which has been also tested through a case of entrepreneurship. The study finds that: (1) there are 15 factors influencing entrepreneurial learning from failure that play different hierarchical roles; (2) the entrepreneurs’ self-efficacy, as a key influencing factor of entrepreneurial learning from failure, can be cultivated and improved by enriched the entrepreneurs’ successful career experience. In addition, emotion regulation after the entrepreneurial failure is also a key influencing factor of the entrepreneurial learning from failure and the emotion management is deemed as an important part of entrepreneurship education; (3) the entrepreneurial education may affect the entrepreneurship learning from failure indirectly by affecting the entrepreneurs’ self-efficacy; (4) the economic conditions, the policy support, the industry characteristics and the cultural sensemaking of failure are the macro factors that may affect the entrepreneurship learning from failure.

## Introduction

According to Drucker, the most important factor of success is the ability of learning from previous mistakes and applying what have been learned in a more effective way. Nevertheless, most people pay more attentions to successful enterprises both in entrepreneurship research and entrepreneurship practice due to anti-failure bias (Crane and Sohl, 2004; Razmus and Laguna, 2018), rarely from failures. There are few practice and research on the failure of entrepreneurship and subsequent learning after failure because of lack of emphasis on learning from failure and lack of a correct understanding of how to treat these failures. Recently, researchers have found that failure is also an important resource filled with skills and knowledge to update entrepreneurial activities, thus help entrepreneurs reduce their uncertainty and expand their scope of seeking new business opportunities (Mantere et al., 2013; Khelil, 2016). Meanwhile, some researchers have focused on how entrepreneurs can benefit and learn from failure (Gong et al., 2009; Benson and Han, 2011). As proposed by Cope (2011), failure can expand the entrepreneurs’ scope of potential behaviors by correcting their ineffective practices and improving their skills and knowledge. Gong et al. (2009) also believed that entrepreneurs should not only learn from other successful entrepreneurs but learn from their own failure. Therefore, the study of entrepreneurial learning from failure is of important theoretical value and practical significance.

The existing studies focus on the entrepreneurial learning from failure from three perspectives. First of all is the learning style. The mode of entrepreneurial learning from failure refers to the way how the entrepreneurs learn from their entrepreneurial failure, including the influence of entrepreneurial failure on the choice of learning style (Politis, 2005) and the relationship between the style of entrepreneurial learning from failure and the learning content (Cope, 2011). The second perspective is the research on the content of entrepreneurship learning from failure (Shepherd et al., 2009b; Wang and Chugh, 2014), the studies on which are diversified, including self-learning, business learning, network and relationship learning and new enterprise management learning. Among others, the self-learning is the core of entrepreneurship learning from failure (Cope, 2011; Jenkins et al., 2014). Pittaway and Thorpe (2012) proposed that learning from failure includes the internal learning and the external learning, the former of which refers to the knowledge of creating, managing, and closing enterprises while the later refers to the opportunity identification and entrepreneurship awareness. The last one is about the mechanism of entrepreneurial learning from failure (Shepherd, 2004; Cardon et al., 2011). At present, limited researches have been carried out about the mechanism of entrepreneurial learning from failure, mostly focusing on entrepreneurial performance and sustainable entrepreneurship in the study of outcome variables (Steffens et al., 2009; Carsrud and Brännback, 2011; Gorgievski et al., 2014). To sum up, the researches on the internal structure of entrepreneurial learning from failure are still uncertain. It is unclear why the learning occurs after entrepreneurial failure and how to carry out such entrepreneurial learning from failure. Some achievements have been made in the existing research, but the research perspectives are more scattered, indicating a relatively definite system not yet established.

On the basis of literature review, this paper has sorted out the influencing factors of entrepreneurial learning from failure, finding that there are 24 factors that may influence the entrepreneurial learning from failure. Upon analysis of the expert panel, 15 factors are finally formed. In reference to the Interpretive Structure Model (ISM) method of system dynamics (Warfield, 1978; Sushil, 2012), the structure chart of the mutual relationship is thus obtained and the multi-level structure of the factors influencing entrepreneurial learning from failure is finally drawn. On this basis, this paper further revises the research model in respect of the influencing factors of entrepreneurial learning from failure through a case study.

This paper makes a number of significant contributions. First of all, an ISM of system dynamics is applied to study the factors affecting the entrepreneurial learning from failure, which is a beneficial attempt of ISM method in the field of entrepreneurial research and provides a new research tool for entrepreneurial research. Secondly, this paper explores the antecedent variables affecting the entrepreneurial learning from failure, which makes it possible to clarify the mechanism of entrepreneurial learning from failure. Thirdly, the paper provides a clear path for entrepreneurs to improve their capacity for learning from failure, in which the entrepreneurs’ self-efficacy and emotion regulation deserve more attentions.

## Literature Review

In early studies, the failure was considered to be actively avoided because it is costly and unpleasant (Baron and Markman, 2003), and may cause a vicious cycle of frustration and decline (Artinger and Powell, 2016). However, the entrepreneurial failure has been recently recognized valuable as an important resource of developing skills and knowledge, improving learning and thus raising their entrepreneurial opportunity (Cardon et al., 2011). The existing researches show that the factors affecting entrepreneurial learning from failure can be categorized into 12 individual factors, 4 enterprise factors, and 8 environmental factors, as shown in Table 1.

**Table 1:** Identified factors of entrepreneurial failure learning.

Notation	Factors	Type of research	Relationship	References
F1	Entrepreneurship failure experience	Quantitative	Positive	Boso et al., 2018
F2	Critical career experiences	Quantitative	Positive	Politis and Gabrielsson, 2009
F3	Alertness	Quantitative	Positive	Boso et al., 2018
F4	Self-leadership	Qualitative	Positive	Boss and Sims, 2008
F5	Personality traits	Qualitative	Comparison	Cotterill, 2012
F6	Persistence	Qualitative	Comparison	Cotterill, 2012
		Quantitative	Inverted U shaped	Shepherd et al., 2009a
F7	Self-efficacy	Conceptual	Positive	Roxane and Frank, 2014
F8	Confidence	Quantitative	Inverted U shaped	Trevelyan, 2008
F9	Sense of failure	Qualitative	Positive	Oser and Volery, 2012
F10	Emotional costs	Quantitative	Positive	Jenkins et al., 2014
		Qualitative	Negative	Shepherd et al., 2009b
		Conceptual and Qualitative	Case	Cope, 2011
F11	Emotion regulation	Qualitative	Positive	Boss and Sims, 2008
		Quantitative	Positive	Vivianna et al., 2017
F12	Psychological capital	Conceptual	Positive	Roxane and Frank, 2014
F13	Financial costs	Qualitative	Comparison	Cardon et al., 2011
		Conceptual and Qualitative	Case	Cope, 2011
		Qualitative	Negative	Shepherd et al., 2009b
F14	Learning style	Qualitative	Comparison	Vinig and Souren, 2007
F15	Cultural sensemaking	Quantitative	Comparison	Cardon et al., 2011
F16	Failure velocity	Quantitative	Inverted U shaped	Vivianna et al., 2017
F17	Stigma of entrepreneurial failure	Qualitative	Case	Landier and Holmstrom, 2005
F18	Economic conditions	Quantitative	Comparison	Carlos et al., 2016
F19	Social capital	Qualitative	Positive	Anderson et al., 2007
F20	Industry characteristics	Qualitative	Comparison	Macpherson and Holt, 2007
F21	Environment conditions	Qualitative	Comparison	Sapovadia, 2015
F22	Policy support	Qualitative	Positive	Carayannis et al., 2003
		Qualitative	Positive	Sapovadia, 2015
F23	Luck	Qualitative	Comparison	Cotterill, 2012
		Qualitative	Comparison	Liu, 2010
F24	Entrepreneurship education	Qualitative	Positive	Carayannis et al., 2003

### Individual Factors

Individual factors can affect the entrepreneurial learning from failure from two perspectives, one of which is the entrepreneur’s personal factors. Recent researches showed that the entrepreneurial learning from failure would be influenced by the entrepreneurial failure (F_1_) and would exert a positive impact on the performance of new enterprises (Boso et al., 2018). Meanwhile, the critical career experience (F_2_) also plays a significant role in promoting the development of entrepreneurs’ attitudes toward failure (Politis and Gabrielsson, 2009), which would enable individuals more alert to new business opportunities (F_3_) (Boso et al., 2018). Boss and Sims (2008) adopted the context of failure, suggesting that the self-leadership (F_4_) can help those who have experienced failure move toward recovery more easily than those who have not yet been engaged. In the conceptual framework for analysis of failure, Cotterill (2012) proposed that the entrepreneurial personality traits (F_5_) have set up a new venture through entrepreneurial response and the entrepreneurial persistence (F_6_) may remain steadfast in the new venture regardless of failure (Shepherd et al., 2009a). Roxane and Frank (2014) referred five cognitive processes of self-efficacy (F_7_), which may allow individuals to take time to reflect on both his/her past successes and failures, contributing to a progress. Analogously, a positive effect of confidence (F_8_) on entrepreneurial tasks was found for both action and judgment tasks (Trevelyan, 2008; Hogarth and Karelaia, 2012).

On the other hand, the entrepreneurs’ emotional management may also affect their learning from failure. Scholars called for a balanced approach to entrepreneurship education and training by developing a sense of success and a sense of failure (F_9_) in order to help them learn from failure (Oser and Volery, 2012), and further developed a model to reconcile the countervailing effects of failure (Vivianna et al., 2017). In order to delay the business failure, researches also suggested a positive effect in balancing the financial and emotional costs (F_10_) of business failure to promote the overall recovery under some circumstances (Shepherd et al., 2009b), in which the emotional toll is the hardest (Cope, 2011), and then the emotion regulation (F_11_) plays an important role of moderation (Vivianna et al., 2017). Another important factor that cannot be ignored is the psychological capital (F_12_), which is considered playing a moderating role in the relationship between the negative consequences of failure and the positive effects of learning from failure (Roxane and Frank, 2014).

### Enterprise Factors

The factor of enterprise operation may directly affect the entrepreneurial learning from failure. Financial cost pressures (F_13_) are critical to the entrepreneurs’ ability of learning when they fail (Cardon et al., 2011). Especially, the delayed business failure can be financially costly, making it more difficult for the enterprises to recover from the failure (Shepherd et al., 2009b). In addition, the learning style (F_14_) of enterprises also directly affects whether they can learn lessons and continue to start businesses after failure (Vinig and Souren, 2007). The research results suggested that the majority entrepreneurs use accommodative learning style by reliance on practical experience, intuition and imagination (Vinig and Souren, 2007). Some scholars also observed venture failure through the lens of cultural sensemaking (F_15_), proposing that the stigmatization of entrepreneurs at local area would be influenced by the venture failure, which would further affect their attitude and behavior of individuals after their failure (Cardon et al., 2011). In addition, they also focused on failure velocity (F_16_) to understand the entrepreneurs’ learning from failure (Vivianna et al., 2017).

### Environmental Factors

The environmental dimension is an important factor for entrepreneurs. Researches show that different levels of cultural tolerance would affect the stigma of failure (F_17_) in entrepreneurship (Landier and Holmstrom, 2005). Perceived low cultural tolerance is more likely to aggravate the entrepreneurial stigma (Singh et al., 2015), which thus hinders the entrepreneurial activities (Simmons et al., 2014). The combination of fundamental entrepreneurial factors is identified as the driving force for the growth of new businesses under different economic conditions (F_18_) (Carlos et al., 2016). Human and social capital (F_19_), organizational systems, industry characteristics (F_20_) and knowledge network are combined to facilitate or restrict growth (Anderson et al., 2007; Macpherson and Holt, 2007). Sapovadia (2015) referred that a supportive environment condition (F_21_) and policy support (F_22_) for entrepreneurial ventures or act as impediments to its growth.

Meanwhile, the environmental factors may affect the entrepreneurs’ attitudes toward failure in the context of their background, including their immigration, educational and socio-economic background, reputation, and stigma (Cotterill, 2012). Luck (F_23_) is also an important aspect of entrepreneurship practice. Liu (2010) indicated that people tended to over-attribute their own successes to superior skills but failures to bad luck. The learning from failure can be affected and restricted by many factors, two of which deserve our special attentions: the ability to face the failure and the ability to take risks (Cannon and Edmondson, 2001). As one of the most important abilities to undertake risks, the social capital has also attracted the researchers’ attentions. What is particularly mentioned is the influence of entrepreneurial education (F_24_) on the entrepreneurial learning from failure. The entrepreneurial education can improve the entrepreneurs’ effective cognition of entrepreneurship failure and consider failure as a useful learning experience so as to recover from failure and continue to start a business as soon as possible (Carayannis et al., 2003).

## Materials and Methods

### Methods

The ISM refers to a process that transforms unclear and poorly articulated models of systems into visible and well-defined models for many purposes (Farris, 1975; Sushil, 2012). It emphasizes that the analysis of things has to be rooted in the collection of realistic materials and the processing and analysis of data. Through theoretical deduction, it extracted the interaction mechanism among the elements of the complex system, and finally formed a theoretical concept (Valmohammadi and Dashti, 2016). ISM has been widely applied to the study of the antecedents of practical problems in the field of management (Feng and Yun, 2010; Muruganantham et al., 2018). This study adopts the method of ISM to probe into the factors influencing entrepreneurial learning from failure and makes conclusions by a hierarchical topology figure for intuitively understanding the structure of system factors. Moreover, a typical case study is conducted to analyze the key factors of entrepreneurial failure learning and test the rationality of the model.

We carry out an ISM study on the causes of entrepreneurial learning from failure through four steps: firstly, to extract the influencing factors widely on the basis of problem analysis; secondly, to screen out important influencing factors with the help of an expert panel; thirdly, to design the relationship structure of factors by using statistical software and other technical tools; fourthly, to carry out hierarchical processing to form a multi-level conceptual model of interpretative structural system. With this method, the combined elements and their relationship in complex systems can be clarified to facilitate understanding and control.

### Analysis

Scholars have explained the factors influencing the entrepreneurial learning from failure from different perspectives and levels. Due to the difficulty in identifying all the factors through existing research methods (e.g., questionnaire survey and case study), we have concluded 24 factors influencing the entrepreneurial learning from failure (as shown in Table 1) through a comprehensive literature review in the first step.

In order to clarify the important factors affecting the entrepreneurial learning from failure and the relationship among them, an expert panel was established to identify these factors in the second step, composed of 15 members including three researchers who teach entrepreneurship theory in the universities, eight entrepreneurs, two government staff and two experts from the incubator. First of all, we made clear to all members the conception of all factors and entrepreneurial learning from failure. Fifteen experts were requested to evaluate back-to-back whether 24 factors had an impact on the entrepreneurial learning from failure. Moreover, they can write down the factors not mentioned in the literature if they had different opinions. The results showed that 13 factors were unanimously agreed by more than 10 experts and 2 additional factors (the failure expectation and the family’s support) were respectively agreed by more than two thirds of experts (Kuo et al., 2010; Valmohammadi and Dashti, 2016). Upon discussion between the experts and the entrepreneurs, we removed 11 factors and added 2 factors, finally confirmed 15 factors. The purpose of removing the unimportant factors was to help entrepreneurs grasp the key factors of entrepreneurial learning from failure after their business failure.

**Table 2:** Pair-wise comparison of the factors.

O	O	O	O	O	O	O	O	O	V	O	O	O	O	Entrepreneurship failure experience (O_1_)
O	O	A	O	O	O	O	O	O	V	O	O	O	Entrepreneurship education (O_2_)	
X	A	A	A	A	O	O	V	O	O	O	O	Environment conditions (O_3_)		
O	O	O	O	O	O	O	O	X	A	A	Self-efficacy (O_4_)			
O	O	O	O	O	O	O	O	O	X	Social capital (O_5_)				
O	O	O	O	O	O	O	O	O	Psychological capital (O_6_)					
O	O	O	O	A	A	A	A	Emotion regulation (O_7_)						
O	O	O	O	O	O	X	Expectation of failure (O_8_)							
A	O	O	O	O	O	Sense of failure (O_9_)								
O	O	O	O	O	Personality traits (O_10_)									
O	O	O	O	Family support (O_11_)										
O	O	X	Economic conditions (O_12_)											
O	O	Policy support (O_13_)												
O	Industry characteristics (O_14_)													
Cultural sensemaking (O_15_)														

**Table 3:** Reachable matrix of the factors.

No	O_1_	O_2_	O_3_	O_4_	O_5_	O_6_	O_7_	O_8_	O_9_	O_10_	O_11_	O_12_	O_13_	O_14_	O_15_
O_1_	1	0	0	0	0	1	0	0	0	0	0	0	0	0	0
O_2_	0	1	0	0	0	1	0	0	0	0	0	0	0	0	0
O_3_	0	0	1	0	0	0	0	1	0	0	0	0	0	0	1
O_4_	0	0	0	1	0	0	1	0	0	0	0	0	0	0	0
O_5_	0	0	0	1	1	1	0	0	0	0	0	0	0	0	0
O_6_	0	0	0	1	1	1	0	0	0	0	0	0	0	0	0
O_7_	0	0	0	1	0	0	1	0	0	0	0	0	0	0	0
O_8_	0	0	0	0	0	0	1	1	1	0	0	0	0	0	0
O_9_	0	0	0	0	0	0	1	1	1	0	0	0	0	0	0
O_10_	0	0	0	0	0	0	1	0	0	1	0	0	0	0	0
O_11_	0	0	1	0	0	0	1	0	0	0	1	0	0	0	0
O_12_	0	0	1	0	0	0	0	0	0	0	0	1	1	0	0
O_13_	0	1	1	0	0	0	0	0	0	0	0	1	1	0	0
O_14_	0	0	1	0	0	0	0	0	0	0	0	0	0	1	0
O_15_	0	0	1	0	0	0	0	0	1	0	0	0	0	0	1

Thirdly, the relationship between influencing factors were discussed by experts, and we adopted the majority of opinions after identification of the 15 factors. Experts were asked to conduct a pair-wise comparison of 15 factors. The factors were denoted O_i_, where i = 1, 2, …, 15, as shown in Table 2. When judging the relationship between the factor O_i_ and O_j_, the experts were asked to select from one of the following four types:

• Type V: factor O_i_ has a direct effect on factor O_j_
• Type A: factor O_j_ has a direct effect on factor O_i_
• Type X: factor O_i_ and a reciprocal effect on factor O_j_
• Type O: factor O_i_ and factor O_j_ are mutually unrelated.

It is relatively easy for experts to make a consistent judgment on the relationship among the 15 factors. The final consensus on the pair-wise comparison is shown in Table 2.

Fourthly, we used ISM method to divide the 15 important factors that may affect the entrepreneurial learning from failure into different levels and thus get an intuitive interpretation model. On the basis of Table 2, this study used a 15 × 15 square matrix to express the logical correlation among the important factors affecting the entrepreneurial learning from failure, forming an adjacency matrix A that covers any two or two elements in the whole influencing factors system. Among them, a_ij_ refers to the elements in line i and column j of a square matrix (i, j = 1, 2,…,15), indicating the relationship between the influencing factors O_i_ and O_j_. “1” in row i and column j indicates that Factor i has an effect on Factor j. Besides, as the influencing factors of complex systems are not directly related, we use the reachability matrix to obtain and master the relationship between the direct and indirect effects of one factor on other factors, as well as the transitive representation of each factor.

The reachable matrix (R) is mainly used to express the transfer relationship between the direct or indirect effects of the influencing elements. The r_i_ can reach r_j_ by the distance of Unit 1, and r_j_ can still reach the next influencing factor by the distance of Unit 1. Based on the sum of adjacent matrix A and unit matrix I, A+I = B is formed. Boolean algebraic power operation is carried out on B, and the reachable matrix R can be obtained without producing a new “1” in the operation result. It shows all the direct and indirect relationships among the factors of the entrepreneurial learning from failure. By using the analytic logic path of reachable matrix (Kuo et al., 2010; Kannan et al., 2014), based on the analysis result of adjacency matrix, we use Matlab to calculate B^n^ until the calculation satisfies B^n-1^ = B^n^ (*n* = 2). Table 3 shows the reachability matrix R of each influencing factor.

The procedure for deriving the final multilevel structure hierarchy is shown in Table 4. R(O_i_) refers to the reachable set of O_i_ and C(O_i_) represents the precedence set of O_i_. When R(O_i_) = R(O_i_)∩C(O_i_), R(O_i_) is placed in a set corresponding to the level and excluded in the analysis of subsequent levels (Hussain et al., 2016; Thirupathi and Vinodh, 2016). We divide these 15 factors into different levels by this method. The result shows that the factors can be partitioned into four levels as follows (Table 4):

• Level 1: 4, 7
• Level 2: 5, 6, 8, 9, 10
• Level 3: 1, 2, 3, 15
• Level 4: 11, 12, 13, 14

## Results

**FIGURE 1:**
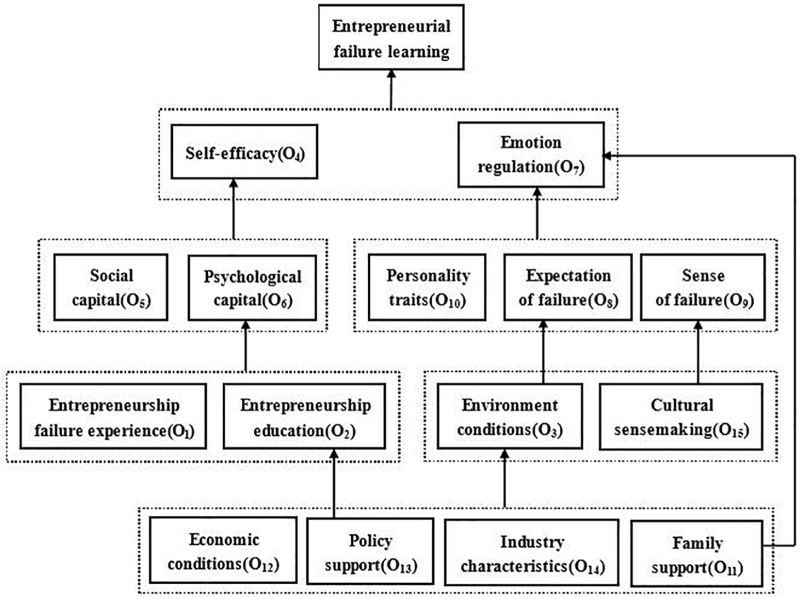
Multilevel structure hierarchy of the factors.

After ISM analysis, the relationships among these factors were illustrated by using a multilevel structure hierarchy chart, as shown in Figure 1, to guide activities of the entrepreneurial learning from failure. Results of the analysis are summarized as follows:

Special attentions need to be paid to the self-efficacy (O_4_) of entrepreneurs, which may directly affect the entrepreneurial learning from failure. The results also show that the social capital (O_5_) and the psychological capital (O_6_) can indirectly affect entrepreneurial learning from failure through self-efficacy; in other words, the social capital and the psychological capital of entrepreneurs play an important role in improving the self-efficacy. On the other hand, the entrepreneurs’ failure experience (O_1_) and the entrepreneurship education (O_2_) can directly affect entrepreneurs’ psychological capital. The entrepreneurship education has to more concern the construction of entrepreneurs’ psychological capital.

The emotion regulation (O_7_) plays a crucial role in the entrepreneurial learning from failure. We have demonstrated that how the entrepreneurs manage their emotions is closely related to how they learn after a failure. The personality traits (O_10_), the expectation of failure (O_8_), and the sense of failure (O_9_) all have exerted influences on the emotion regulation. The environment conditions (O_3_) can affect the entrepreneurs’ expectations on failure and the culture sensemaking (O_15_) can affect the sense of failure caused by entrepreneurship.

In addition, the entrepreneurial learning from failure is indirectly affected by the macro environment, including the economic conditions (O_2_), the policy support (O_13_), the industrial characteristics (O_14_) and the family support (O_11_). We also find that the policy support can directly affect the entrepreneurship education.

## Case Study

In order to test the rationality of the model, we chose a typical case to study the key factors affecting entrepreneurial learning from failure. There are three main reasons. Firstly, all three entrepreneurs in this case had entrepreneurial failure experience during entrepreneurship. Secondly, they recovered from their failures and continued to start their own businesses. Thirdly, the company belongs to the high-tech industry, which is considered to have a high failure rate of entrepreneurship (Gong et al., 2009). Therefore, this case is representative for the study of factors affecting entrepreneurial learning from failure.

The case company which is engaged in the development and sales of educational software in China was founded in recent 5 years. Now it has built up its business relations with more than 30 universities in Asia by providing the original educational development software. Its sales contracts have exceeded one hundred thousand dollars in the first year with its sales growing at an average rate of more than 120% annually.

**Table 4:** Interpretive Structure Model analysis of the factors.

Level	O_i_	R(O_i_)	C(O_i_)	R(O_i_)∩ C(O_i_)
1	1	1,6	1	1
	2	2,6	2,13	2
	3	3,8,15	3,11,12,13,14,15	3,15
	4	4,7	4,5,6,7	**4,7**
	5	4,5,6	5,6	5,6
	6	4,5,6	1,2,5,6	5,6
	7	4.7	7,8,9,10,11	**4,7**
	8	7,8,9	3,8,9	8,9
	9	7,8,9	8,9,15	8,9
	10	7,10	10	10
	11	3,7,11	11	11
	12	3,12,13	12,13	12,13
	13	2,3,12,13	12,13,14	12,13
	14	3,14	14	14
	15	3,9,15	15	15
2	1	1,6	1	1
	2	2,6	2,13	2
	3	3,8,15	3,11,12,13,14,15	3,15
	5	5,6	5,6	**5,6**
	6	5,6	1,2,5,6	**5,6**
	8	8,9	3,8,9	**8,9**
	9	8,9	8,9,15	**8,9**
	10	10	10	**10**
	11	3,11	11	11
	12	3,12,13	12,13	12,13
	13	2,3,12,13	12,13,14	12,13
	14	3,14	14	14
	15	3,9,15	15	15
3	1	1	1	**1**
	2	2	2,13	**2**
	3	3,15	3,11,12,13,14,15	**3,15**
	11	3,11	11	11
	12	3,12,13	12,13	12,13
	13	2,3,12,13	12,13,14	12,13
	14	3,14	14	14
	15	3,15	15	15
4	11	11	11	**11**
	12	12,13	12,13	**12,13**
	13	12,13	12,13,14	**12,13**
	14	14	14	**14**

*Bold values represent the factors of each level in the interpretative structural model.*

The company has three entrepreneurship partners. In order to protect the privacy of participants involved, this paper uses A, B, and C respectively instead of their names. After graduation from a university in China, A joined an education software company. Seven years later, A became the regional sales director responsible for the sales and maintenance of university education software in China. With sound social capital and network resources (social capital) in this industry, he has established a good professional reputation (professional experience) in the industry. In the 8th year, A left the original software company and started an educational software company by himself. In the company, he is responsible for technology and product research and development, sales and management of the founding team. Partner B had three entrepreneurial experiences before joining the company. He has been engaged in entrepreneurship projects of maternal and infant e-commerce, early childhood education and online courses. With profound Internet experience and clear understanding of the customers’ needs, he was an industry expert in education informatization with strong self-driving force (self-efficacy). Partner C is responsible for the company’s marketing, including the market development and the maintenance of customers in the previous company. At the same time, as a technical partner highlighting rich experience in technology research and development, he is also acting as the technical architect of the company, indicating a good social capital from investors. They all suffered from failure (entrepreneurship failure) before they became partners.

At the initial stage of the company’s development, disputes arose in respect of the product positioning and the company’s development direction. The three partners could not persuade each other, resulting in conflicts and leading to emotional disorder (emotion regulation). In the process of development, “the company lacked the entrepreneurship education, manpower and expertise in the incubator, as well as work experience, services and business consulting” partner A said. In addition, the entrepreneurship education and the government policies were not sufficient. Partner B believed that the family support should be very important for the recovery from business failure. Partner C especially mentioned that the management of entrepreneurs’ emotions was of great significance and the expectation of failure would affect the learning of entrepreneurial failure.

Respondents highlighted the social capital and the family support, which they believed as important factors for the entire entrepreneurial team. Within the entrepreneurial team, the members’ self-efficacy, communication and emotional adjustment after setbacks are deemed as difficult problems; therefore, considering the above interview case, this article emphasizes that although the factors influencing the entrepreneurial learning from failure have different emphases on different entrepreneurs, they demand strong self-management and psychological control ability to support the entrepreneurship to continue. The discussion of the case is well in line with the explanatory structure model constructed in this paper, and the key factors affecting the entrepreneurial learning from failure show hierarchical characteristics.

## Conclusion

This paper sorted out the influencing factors of entrepreneurial learning from failure and found that 15 factors influencing entrepreneurial learning from failure. By referring to the ISM method of system dynamics (Warfield, 1978; Sushil, 2012), the structure chart of the mutual relationship is thus obtained, and the multi-level structure level of the factors influencing entrepreneurial learning from failure is finally drawn.

In combination with the above research results, this research emphasizes the role of the entrepreneur’s emotion regulation, which directly affects the entrepreneurial learning from failure. The entrepreneurial activity is a great challenge to the entrepreneurs, both physically and mentally, and the ability of controlling emotions is extremely important for the entrepreneurs to get recovered and learn from their failure. This finding is an important extension of the research conclusion of Cope (2011), who emphasized that the entrepreneurial emotion management shall deserve close attention. In addition to the financial cost, the emotional cost is another cost generated from entrepreneurial failure (Shepherd et al., 2009b). In this sense, the entrepreneurship emotion management is an important part of entrepreneurship education, which is different from the previous entrepreneurship education that mostly emphasizes entrepreneurship skills and business models (Honig, 2004; Henry et al., 2005; Neck and Greene, 2011). Besides, we also find in the interview that the entrepreneurs’ emotions, such as loneliness, frustration and stigma, are their daily emotions rather than those only generated after failure. In this sense, the entrepreneurship education institutions need to provide entrepreneurs with spiritual mentors and more professional psychological consultation. At the same time, the entrepreneurs themselves need to conduct effective emotion management and monitoring in entrepreneurial activities to prevent the spread of negative emotions.

This paper also illustrates that entrepreneurs’ self-efficacy is another key influencing factor of the entrepreneurial learning from failure (Karl et al., 2013). According to the three research perspectives of self-efficacy as divided by Bandura (1982), the entrepreneurs with a high sense of self-efficacy choose appropriate tasks, which coincide with their ability from the perspective of behavior. What’s more, this research points out that the greater the possibility of success after failure, the more efforts they will make and the stronger persistence of entrepreneurial behavior will be. From the perspective of the attitude and the degree of effort, people with a high sense of self-efficacy can face up to failure and difficulties more bravely, overcome difficulties to achieve their entrepreneurial goals with their efforts in a more confident manner (Wu et al., 2019). From the perspective of the thinking model of entrepreneurial learning from failure, people can focus on analyzing the causes of failure and solving difficulties actively with a strong sense of self-efficacy, and show excellent behavioral ability and efficiency.

## Discussion

### Implications

This study has three main aspects in theoretical contribution. Firstly, our study sorts out 24 influencing factors of entrepreneurial learning from failure according to literature review, and proposed a hierarchical model of influencing entrepreneurial learning from failure through the expert method based on ISM method of system dynamics. Existing studies have explored a lot of factors affecting entrepreneurial failure learning from three levels, including individual, enterprise and environmental aspects (Shepherd et al., 2009b; Carlos et al., 2016; Vivianna et al., 2017). We carry out a comprehensive research and present an intuitive ISM (as shown in Figure 1) for researchers. Secondly, this paper reveals that self-efficacy and emotion regulation may exert direct impacts on entrepreneurial learning from failure as key factors. Some scholars have also stressed the importance of self-efficacy and emotion regulation (Boss and Sims, 2008; Roxane and Frank, 2014). This provides a new clue to understand the study mechanism of entrepreneurial failure from comprehensive function of self-efficacy and emotion regulation. Furthermore, this study integrates the existing research dimensions and research framework, which is a beneficial exploration of the entrepreneurial learning theory and also provides a possibility for empirical study of entrepreneurial learning from failure.

In practice, our research findings have provided potential implications for entrepreneurs and organizations to build entrepreneurial systems in three aspects. This paper firstly provides a path for entrepreneurs to improve their ability of learning from failure. The entrepreneurs’ self-efficacy and emotion regulation deserve high attention because they may directly affect the entrepreneurial learning from failure. Entrepreneurs should maintain a high level of self-efficacy and regulate their emotions to facilitate the beneficial transformation of entrepreneurial failure (Petrovic et al., 2016). Secondly, the entrepreneurship education may indirectly affect the entrepreneurial learning from failure by affecting the entrepreneurs’ self-efficacy. As the main institution and department of entrepreneurship education, universities, governments and entrepreneurship education institutions are required to provide entrepreneurs with more accurate entrepreneurship services, such as apprenticeship entrepreneurship mentors, which will not only provide business guidance and resource integration, but also emphasize the entrepreneurs’ psychological capital and emotion regulation. Thirdly, economic conditions, policy support, industry characteristics and cultural sensemaking of failure are the macro factors that cannot be ignored (Christiansen, 2006). Especially, the government has to provide vigorous innovation policy support, industry information and public services so as to create a good social atmosphere and environment for entrepreneurial activities.

### Limitation and Future Research

The ISM proposed in this paper integrates and extracts the existing literature. The relationship between different influencing factors is analyzed from the perspective of system theory; besides, the model is revised and expanded by means of the case study method. Although this paper can provide enlightenment on the application of the method of ISM in the field of entrepreneurship to a certain extent, there are inevitably some limitations in this paper, providing the directions of future studies.

First, this paper uses ISM method to study the factors influencing the entrepreneurial learning from failure. Although this method has been partly applied in some field of management (Feng and Yun, 2010; Muruganantham et al., 2018), its applicability in the field of entrepreneurship needs further study. Therefore, researchers can expand the applicability of ISM method in the research of entrepreneurship.

Secondly, the combination with the entrepreneurial learning theory makes a hierarchical judgment of each influencing factor, but fails to conduct a quantitative study. It is still a subjective judgment of the causal relationship between the influencing factors in lack of strong empirical support. Therefore, the conclusion needs more empirical research to provide more evidence support. Especially, self-efficacy and emotion regulation play a key role in influencing entrepreneurial learning from failure (Roxane and Frank, 2014; Vivianna et al., 2017), interaction effect can be considered in future empirical studies.

Thirdly, from the perspective of case study, a single case is selected, which is insufficient to fully explain the model as proposed in this paper. As mentioned above, different approaches of cultural sense-making may exert different effects on individual attitudes and behaviors (Cardon et al., 2011). Therefore, future research should consider more cases from different countries to enrich the conceptual model from the cross-case perspective.

## Informed Consent

The authors state that for this study written informed consent are obtained from all participants. Written informed consent was also obtained from the three entrepreneurs for the publication of the case study/description. All participants are willing to participate in this study and know the content and process of this study.

## Ethics Statement

Based on the existing literature, this paper studies the factors affecting learning after entrepreneurial failure by using the Interpretive Structural Model (ISM) of systematics. The purpose, content, process and conclusion of our research do not involve ethical and moral issues. An ethics approval was not required as per applicable institutional and national guidelines.

## Author Contributions

JW participated in the design, drafting of the early version, and revising of the article. YC participated in model and data analysis, drafting of the early version, and revising of the article. JZ participated in the design and revise of the article. YG checked the method.

## Conflict of Interest Statement

The authors declare that the research was conducted in the absence of any commercial or financial relationships that could be construed as a potential conflict of interest.
